# Outcomes of Intravitreal Dexamethasone Implant in the Treatment of Recalcitrant Diabetic Macular Edema

**DOI:** 10.4274/tjo.28863

**Published:** 2017-10-27

**Authors:** Dorukcan Akıncıoğlu, Murat Küçükevcilioğlu, Ali Hakan Durukan, Seçkin Aykaş, Önder Ayyıldız, Fazıl Cüneyt Erdurman

**Affiliations:** 1 Gülhane Training and Research Hospital, Ophthalmology Clinic, Ankara, Turkey

**Keywords:** dexamethasone, recalcitrant, diabetic, implant

## Abstract

**Objectives:**

To investigate the efficacy and safety of intravitreal dexamethasone (OZURDEX®) implantation in patients with recalcitrant diabetic macular edema.

**Materials and Methods:**

This is a retrospective non-randomized study of patients who underwent intravitreal dexamethasone implantation for recalcitrant diabetic macular edema. Main outcome measures included changes in best corrected visual acuity (BCVA), central macular thickness (CMT), and incidence of ocular side effects.

**Results:**

Fifty-seven eyes of thirty-eight patients (20 females, 18 males; mean age 65±7 years) were included in the study. The mean hemoglobin A1c level was 7.9±1.7%. Before entering the study, patients had undergone 5.71±3.40 anti-vascular endothelial growth factor (anti-VEGF) and 3.44±2.46 intravitreal triamcinolone acetonide injections. The mean duration of diabetes and diabetic macular edema was 17.2±6.4 years and 60.2±17.6 months, respectively. At baseline, mean CMT was 506.76±166.74 µm, and the mean BCVA was 0.68±0.38 LogMAR. Mean CMT significantly decreased to 341.36±146.26 µm (p<0.001), 324.41±114.58 µm (p<0.001), and 384.82±151 µm (p<0.001) at 1, 3, and 4 months of follow-up and increased again to 462.29±152.87 µm at 5 months. Sixteen eyes (28%) received second injections after mean of 7.4±2.3 months and mean CMT was again significantly decreased at 7, 8, and 9 months. Significant improvement in mean BCVA (0.54±0.41 LogMAR; p<0.001) occurred only at 1 month after implantation. However, subgroup analysis revealed significant BCVA improvement in the pseudophakic group at 1, 3, and 4 months. Among phakic patients, 50% showed cataract progression and 28% had elevated intraocular pressure increase which was managed medically.

**Conclusion:**

Intravitreal dexamethasone implantation was effective for the first 4 months in eyes with recalcitrant diabetic macular edema. However, it is hard to displace anti-VEGF agents as first-line therapy due to steroid-related complications.

## INTRODUCTION

Diabetic macular edema (DME) causes reduced central vision due to swelling or thickening of the retinal tissue in the foveal region.^1^ Vision loss is seen in up to 33% of eyes with DME if untreated,^2^ and 6.8% of people with diabetes demonstrate DME.^3^ Lack of improvement solely with strict glycemic control gave rise to serial clinical trials, and laser photocoagulation is considered to be the gold standard based on the Early Treatment Diabetic Retinopathy Study (ETDRS).^4^ Although this study showed that focal laser treatment reduced the risk of visual loss in patients with DME by 50%, visual field defects and subjective visual complaints were considered a serious problem because focal laser treatment was based on disrupting leaking vessels to reduce retinal thickness. Another study revealed increased cytokine levels in the posterior segment causing disruption of blood-retina barrier and leading to edema.^5^ Therefore, targeted therapy was regarded as the new choice. Several studies supported ranibizumab as a favorable alternative to laser therapy. RESTORE provided long-term safety and efficacy data for ranibizumab (Lucentis™, Genentech, San Francisco, CA, USA),^6^ RISE and RIDE reported favorable results in subjects with clinically significant macular edema,^7^ and the Diabetic Retinopathy Clinical Research Network study offered long-term outcomes of laser treatment for DME in combination with anti-vascular endothelial growth factor (VEGF) agents.^8^ Moreover, the BOLT study showed significant visual gain with bevacizumab (Avastin®, Genentech, San Francisco, CA, USA) in comparison to laser therapy alone.^9^ The VEGF-Trap molecule was later designed to decrease VEGF levels more effectively. Two randomized clinical trials (VIVID and VISTA) comparing aflibercept (EYLEA®; Regeneron pharmaceuticals, Inc., Tarrytown, NY, USA) with laser therapy demonstrated its superior efficacy and safety compared to laser therapy.^10^ Nevertheless, ranibizumab is not effective in up to 23% of patients, who are referred to as non-responders.^11^

As DME is a consequence of vascular leakage or proliferation triggered by inflammation, anti-VEGF coverage alone may not be sufficient. Increased concentrations of other pro-inflammatory molecules also mediate its pathophysiology; therefore, corticosteroids, which downregulate many inflammatory molecules including VEGF, are used to restore the blood-retina barrier. A comparative study revealed that ranibizumab with prompt or delayed laser photocoagulation is superior to intravitreal triamcinolone acetonide (IVTA) with laser, especially in phakic eyes, due to the well-known adverse effects of TA.^12^ Moreover, the combination of IVTA and anti-VEGF injections is not superior to anti-VEGF monotherapy.^13^ A highly potent corticosteroid, dexamethasone, failed to resolve DME in a pilot series of single intravitreal injection.^14^ This failure was attributed to the short half-life of intravitreal injections, leading to the development of an implant to provide sustained release.^15^ The MEAD study, which pooled the data of 2 randomized, multicenter, masked, sham-controlled, phase 3 clinical trials to evaluate the dexamethasone biodegradable implant (OZURDEX®, Allergan, Irvine, CA, USA), demonstrated at least 15-letter improvement in best corrected visual acuity (BCVA) from baseline in 22.2% patients receiving the 0.7 mg implant.^16^ In the currrent study, we aimed to evaluate the effects of a single or two consecutive intravitreal dexamethasone (IV-DEX) implants in eyes with recalcitrant DME.

## MATERIALS AND METHODS

We retrospectively reviewed the records of 48 patients who underwent IV-DEX injection due to recalcitrant DME between January 2014 and June 2015 at Gülhane Training and Research Hospital, Ophthalmology Clinic. The protocol was in accordance with the principles of the Declaration of Helsinki and ethics committee approval was obtained. Of the 48 patients, 10 were excluded due to missing data or follow-up, which yielded 38 patients for the analysis. Patients in the study group had diabetes mellitus for a mean of 17.2±6.4 years, and the mean follow-up for DME was 60.2±17.6 months. Patients with recalcitrant DME who met the following criteria were included: age older than 18 years, at least 1 eye with an initial acuity of 0.3 logarithm of the minimum angle of resolution (LogMAR) or worse due to DME, central foveal thickness (CFT) ≥300 µm on spectral domain OCT, and a minimum follow-up of 6 months post-injections. Recalcitrant DME was defined as persistent macular edema with CFT ≥300 µm, lasting 3 months after at least 3 intravitreal anti-VEGF and 3 IVTA injections with 2 sessions of focal grid laser photocoagulation. Exclusion criteria were intraocular surgery within the last 3 months, vitreoretinal interface pathology in the study eye that could prevent improvement, and any other ocular comorbidity contributing to significantly decreased vision. After a detailed explanation of possible risks and benefits of this drug of choice, informed consent was obtained from all patients. Clinical data regarding type and duration of diabetes mellitus, previous treatments, and HbA1c level were recorded. All patients underwent complete ophthalmologic examination including BCVA using standardized ETDRS charts, tonometry, and anterior segment and fundus examination followed by CFT measurements using Heidelberg SPECTRALIS OCT imaging (Heidelberg Engineering, Heidelberg, Germany).

All patients received IV-DEX in the study eye in the operating room under topical anesthesia, and topical moxifloxacin (VIGAMOX®) eyedrop was prescribed four times daily for seven days after injection. The patients were examined on postoperative day 7 for any sign of infection. After injection, the main outcomes including BCVA, CFT, and intraocular pressure (IOP) were assessed at month 1, 3, and every month thereafter. Primary outcome measures were the changes in BCVA and CFT from baseline.

Foveal thickness changes were analyzed using paired t-test, and Wilcoxon test was used to compare preoperative and postoperative LogMAR visual acuity outcomes.

## RESULTS

**Figure 1:**
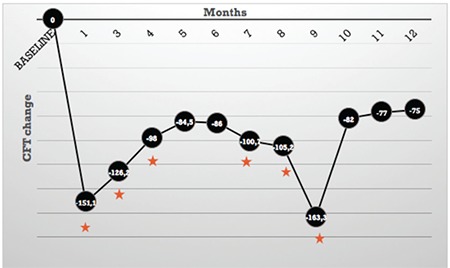
Mean change in central foveal thickness from baseline at each follow-up assessment *Statistically significant, CFT: Central foveal thickness

We analyzed 57 eyes of 38 patients, and baseline characteristics are given in Table 1. All patients had type 2 diabetes, and the mean HbA1c was 7.9±1.7%. Previous intravitreal treatments and laser therapies are given in Table 2. At baseline, the mean CFT was 506.76±166.7 µm, and decreased to 341.36±146.2 µm at 1 month (p<0.0001, paired t-test). Statistically significant decrease was maintained during the following 4 months. The mean decreases in CFT at 5 and 6 months were not significant in comparison to baseline measurements, though they were still below the baseline values. Figure 1 shows the change in CFT. Inter-visit comparisons revealed significant reductions in CFT up to 3 months. Though there was no significant difference between visits at 3 and 4 months (-83.61 µm, p=0.17), measurements at 4 months were still significantly lower when compared to baseline (p<0.0001, paired t-test). Sixteen eyes (28%) underwent second IV-DEX injections with a mean interval of 7.4±2.3 months and CFT decrease was significant at 7, 8, and 9 months. Delay in the second injection was a result of state health policy, which does not approve consecutive injections before 6 months. Twenty-one (36%) eyes remained stable and were followed without injections, while 20 eyes (36%) switched to other agents due to ocular complications, mostly raised IOP.

**Figure 2:**
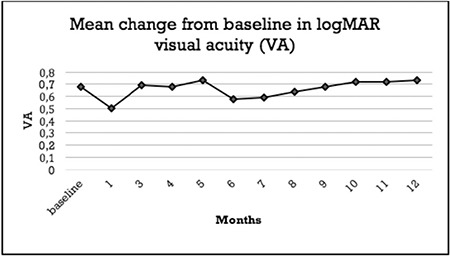
Mean best corrected visual acuity changes from baseline to 12 months VA: Visual acuity

At baseline, the mean BCVA was 0.68±0.38 LogMAR (range 0.10-1.80), and improved significantly only at 1 month (0.54±0.41 LogMAR) (p<0.0001, Wilcoxon). Figure 2 shows the mean BCVA values during follow-up. On the other hand, subgroup analysis revealed interesting results. When we analyzed phakic and pseuodophakic patients separately, the difference in BCVA was significant in the pseudophakic group throughout the 4-month follow-up, whereas there was no significant change in the phakic group.

The cataract progression rate was 50% in phakic eyes, and the mean time for cataract surgery was 5.4±1.1 months from IV-DEX injection. IOP elevation greater than or equal to 10 mmHg from baseline at any visit was seen in 28% of patients, and all patients were managed with medical therapy.

## DISCUSSION

In this study, we aimed to analyze IV-DEX injection as a choice of treatment for recalcitrant DME that is refractory to laser photocoagulation and intravitreal injections of both anti-VEGF and IVTA. There is no consensus or international definition for recalcitrant DME in the English or Turkish literature. Some clinicians consider DME as refractory when there is no improvement following intravitreal injections and some clinicians consider DME as refractory when nonresponsive to maximally applied laser photocoagulation and intravitreal injections of anti-VEGF/TA.^17,18,19^ In the current study, anatomical and functional improvement was significant and maintained for 4 months after injection. Our results are consistent with Zucchiatti et al.^20^ showing improvement in BCVA and CFT as early as the first days after injection and maintained until the fourth month. On the other hand, our results regarding duration of effect are not consistent with the MEAD study, which defined the minimum interval as 6 months.

At 1 month, the mean CFT improved significantly and remained stable throughout the 4-month follow-up. CFT improvement was also significant at 7, 8, and 9 months because second IV-DEX injections were given at a mean of 7.4±2.3 months. As the healthcare regulations of our country prevented us from doing second injections earlier, it is difficult to draw a conclusion about the efficacy of second injections because there is no consistency in the timing of second IV-DEX injections. Moreover, 23% of the study eyes had higher CFT measurements than baseline at 5 months (rebound effect). In this particular group, common characteristics were similar with the rest of the study group and health status was stable. Moreover, their reductions in CFT following injection were similar to the rest of the study group. Therefore, we were unable to identify the exact reason for this rebound effect. Our results regarding CFT are consistent with previous studies demonstrating that statistically significant efficacy continues for 4 months and tends to decrease thereafter.^21,22^

In our series, BCVA improvement was significant only at 1 month after injection, but subgroup analysis revealed significant improvement lasting 4 months in pseudophakic eyes. Nonsignificant BCVA improvement in general may be due to cataract progression, which was noted as early as the 3-month follow-up.

As all of our patients were previously treated, tissue integrity was already compromised. It is known that the shorter the duration of DME, the lower the chance of irreversible damage to the retinal structures.^23^ In addition, Guigou et al.^24^ suggest previous treatments as a negative factor due to irreversible damage to the retinal structure in macular edema. Contrary to clinical trials, there were no treatment-naive eyes in the current study, with the study eyes having a mean of 5.71±3.40 intravitreal anti-VEGF and 3.44±2.46 IVTA injections, and a laser treatment rate of 69.3% before IV-DEX injection. This might explain why we did not achieve significant improvements in BCVA.

## CONCLUSION

Our experience regarding IV-DEX implants in recalcitrant DME cases revealed a good efficiency. Nevertheless, it seems unlikely to displace anti-VEGF agents as first-line therapy due to steroid-related complications.

## Figures and Tables

**Table 1:**
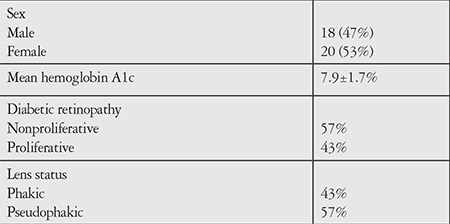
Baseline characteristics of the study population

**Table 2:**
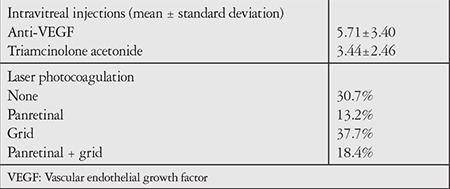
Previous treatments for diabetic macular edema
